# Deciphering the synergistic mechanism of a novel flavonoid-antioxidant combination for asthma by combining systems pharmacology and experimental validation

**DOI:** 10.1371/journal.pone.0346165

**Published:** 2026-03-30

**Authors:** Ziyi Chen, Xinling Ren, Xiaoyu Liu, Lifeng Tian, Yun Huang, Aiping Lyu, Wei Zhou

**Affiliations:** 1 State Key Laboratory of Respiratory Disease Shenzhen University Division, Shenzhen Key Laboratory of Allergy & Immunology, Shenzhen University School of Medicine, Shenzhen University, Shenzhen, China; 2 Department of Respirology & Allergy, Third Affiliated Hospital of Shenzhen University, Shenzhen University, Shenzhen, China; 3 Institute of Brain Science and Brain-inspired Research, Shandong First Medical University & Shandong Academy of Medical Sciences, Jinan, Shandong, P.R.China; 4 Department of Respiratory and Critical Care Medicine, Shenzhen University General Hospital, Shenzhen, China; 5 School of Chinese Medicine, Hong Kong Baptist University, Kowloon, Hong Kong; Guangdong Nephrotic Drug Engineering Technology Research Center, Institute of Consun Co. for Chinese Medicine in Kidney Diseases, CHINA

## Abstract

Asthma is a multifaceted disorder characterized by an intricate interplay of various pathophysiological processes, including airflow obstruction, bronchial hyperresponsiveness, and underlying inflammation. This complexity encompasses a multitude of genetic, molecular, and environmental factors, making it challenging to identify effective strategies for prevention and treatment. Research into the pathogenesis and treatment of asthma continues in the pursuit of more comprehensive and targeted therapeutic approaches. The asthma pathology has been shown to be influenced by the flavonoid 7,4′- Dihydroxyflavone (74DHF), which is extracted from the herb Rhizoma Glycyrrhizae (Gancao), while taking the antioxidant ascorbic acid (VC) daily through food or supplements contributes to the treatment of asthma related to immunomodulatory regulation. However, the potential molecular or systemic mechanism of the combination of 74DHF and VC in treating asthma has yet to be fully understood, despite the demonstrated individual benefits of each compound. The present study employed a systems pharmacology strategy, combining target identification, network analysis, data integration, Gene Ontology (GO) enrichment analysis, pathway analysis, and in vitro experimental validation to uncover the synergistic pharmacological mechanisms of 74DHF-VC combination for asthma therapy. The results identified 153 and 308 targets for 74DHF and VC, 37 overlapping targets and 20 hub targets among 74DHF, VC, and asthma. Based on the GO and pathway analysis, 10 optimal common GO processes and 10 key canonical pathways were enriched, which were closely related to the combination therapy of 74DHF and VC against asthma. In addition, the in vitro experiments validated that 74DHF and VC had synergistic effects on anti-inflammation and fibrosis. The combined treatment reversed the LPS induced inflammation in macrophages and TGF-β induced cell migration and fibrosis in lung epithelial cells. The current study not only successfully explains the synergistic mechanisms underlying the efficiency of 74DHF and VC against asthma but also proposes a viable and promising strategy to fasten the process of combination treatment.

## Introduction

Asthma is characterized by intermittent bronchospasm induced wheezing and shortness of breath [1]. It is an airway disease with airway inflammation, hyper-responsiveness, and mucus hypersecretion. With a global impact on 339 million individuals, asthma carries significant socioeconomic implications [2]. Nevertheless, inadequate treatment remains prevalent. It is crucial to emphasize the criticality of a continuous cycle involving evaluation of asthma control and risk factors, as well as adjustment of medications. A more comprehensive comprehension of these aspects will pave the way for increasingly targeted and efficacious treatment alternatives.

Some clinical available asthma drugs have been used in managing airway obstruction and inflammation such as β2-Adrenoceptor agonists, glucocorticoids, leukotriene receptor antagonists, theophylline, and anticholinergics. However, some side effects were frequently caused [3]. Long-acting inhaled β(2)-agonists and corticosteroids are effective in asthma treatment, but some patients may still experience poor control of disease progression [4]. Leukotriene receptor antagonists can be used in conjunction with inhaled corticosteroids, but their efficacy is lower than that of long-acting beta2 agonists in patients aged over 12 [5]. Despite the safety of theophylline at low serum concentrations the narrow therapeutic range and overdose risk raise concerns [6, 7]. Existing drug options can successfully ameliorate the majority of patients with mild to moderate asthma, but there is a notable lack of adherence to treatment guidelines among patients and healthcare providers. Thus, exploring alternative therapies such as combination drugs is likely to complement the current treatment options and overcome the limitations of conventional drugs.

Natural bioactive ingredients show promise as agents against asthma [8]. Gancao (Rhizoma Glycyrrhizae) is a well-known medicine-food herb that has been used extensively in treating inflammatory diseases, including asthma [9,10]. One of its bioactive components, the flavonoid 7,4’- Dihydroxyflavone (74DHF), has been found to inhibit mucin 5 AC (MUC5AC) gene expression through regulating nuclear factor-κB (NF-κB), signal transducer and activator of transcription 6 (STAT6), and histone deacetylase 2 (HDAC2), thereby alleviating excess mucus hypersecretion in asthma [11]. The *in vitro* study demonstrated that 74DHF inhibits the expression of the memory T helper 2 (Th2) cell transcription factor GATA-binding protein 3 (GATA-3) and reduces interleukin 4 (IL-4) mRNA levels [12]. In addition, 74DHF decreases Th2 responses induced by antigens, such as Th2 cytokine, antigen-specific immunoglobulin E (IgE) production, and airway inflammation in a murine model [12]. Therefore, combining 74DHF with existing medications or bioactive compounds may strengthen the efficacy and reduce adverse effects for asthma therapy.

The antioxidant ascorbic acid (VC) is commonly taken in food and modern medicine to address various conditions related to oxidative stress, inflammation, and immunoregulation [13]. The therapeutic effects of VC has been demonstrated in various respiratory diseases including asthma [13]. Research has reported that administering VC for 16 days decreased IL-4 levels in 1-fluoro-2,4-dinitrobenzene (DNFB)-sensitized BALB/c mice [14]. In a guinea pig asthma model, VC was shown to prevent the accumulation of inflammatory cells in the bronchoalveolar lavage fluid (BALF) and increase cyclic guanosine monophosphate (cGMP) levels, suggesting that VC is effective for airway relaxation and symptom reduction in asthma [15]. Although excessive intake of VC is not typically life-threatening, it can lead to various adverse effect and unexpected health concerns, such as stomach discomfort and kidney stones. Hence, we hypothesize that the combination of 74DHF and VC contributes to the effective control of asthma.

Systems pharmacology has emerged as a holistic and powerful tool to elucidate and validate the complex interactions between small molecules and biological systems based on computational and experimental approaches [16]. Previous studies have successfully employed systems pharmacology-based strategy to identify potential active compounds, targets, compound-target-disease-pathway relationships, and therapeutic effects of phytomedicine for various diseases [17–19]. In the current study, an integrated systems pharmacology framework was developed to systematically elucidate the synergistic mechanisms of 74DHF and VC against asthma. Extensive data mining was conducted to identify potential targets of 74DHF, VC, and asthma-related targets. The common target and the compound-target interaction network of 74DHF and VC were established to uncover the pharmacological synergistic mechanisms of them. Venn diagram and protein-protein interaction (PPI) network analyses were performed to investigate the intersecting hub targets among asthma, 74DHF, and VC. Gene Ontology (GO) analysis and compound-pathway Sankey Bubble chart were used to enrich the biological processes (BP), cellular component (CC), molecular function (MF), and related pathways of 74DHF and VC against asthma. Last but not leaset, the cellular experiments were employed to confirm the anti-inflammation and anti-fibrosis effects from the 74DHF and VC.

## Materials and methods

### Collection of putative targets for 74DHF, VC and asthma

In this study, we identified the putative targets of 74DHF and VC using four comprehensive databases including the Traditional Chinese Medicine Systems Pharmacology (TCMSP) [20], Herbal Ingredients' Targets Platform (HIT) [21], PubChem [22], and PharmMapper [23]. The candidate targets were obtained by screening the above databases and removing the duplicate targets. The diseases corresponding targets were searched using “asthma” as the keywork in the GeneCards database [24]. The UniProt Database was applied to transform the obtained target protein name into their target protein gene name for data standardization [25].

### Venn diagram and network visualization

To elucidate the synergistic action mechanism of 74DHF and VC against asthma, the custom Venn diagram was involved to identify the intersected targets [26]. The compound-target (C-T) network of 74DHF and VC was established to visualize their complicated interactions and the common targets. The protein-protein interactions (PPI) network was obtained by employing the STRING platform to uncover the complex relationships among the intersected genes of 74DHF, VC, and asthma [27]. The C-T and PPI networks were displayed by the Cytoscape 3.6.0 and the topological parameters were determined using the network analyzer plug-in [28]. In C-T network, the nodes represent target genes, and the edges represent the interactions between compounds and targets. In PPI network, each node is target gene, and an edge represents that two genes interact with each other. A node with a larger degree has a more significant impact on the PPI network.

### Gene ontology and pathway analysis of intersected targets

BP, CC, MF, and pathway analysis were conducted to investigate the biological relevance and functional pathways of the intersected hub targets of 74DHF, VC, and asthma. Enrichment results were processed and visualized using the ClueGO Cytoscape plugin [28] to annotate intersected target genes and provide insights into biological processes, cellular component, and molecular function of functional groups. Additionally, the KEGG pathway analysis was employed to interpret the molecular pathways involved in the combination of 74DHF and VC for asthma therapy [28].

### Cell Culture and CCK8 assay

The human bronchial normal cells (BEAS-2B) and macrophage-like cells RAW264.7 were obtained from the American Type Culture Collection (ATCC). The culture was kept in DMEM with 10% FBS at 5% CO2 humidified incubator (37°C). Routine passaging was performed at a 1:3 ratio. Experiments were conducted using cells that reached 90% confluence. We employed recombinant transforming growth factor beta 1 (TGF-β1, Peprotech, 100−21), lipopolysaccharide (LPS, Solarbio, L8880), IL4 (Peprotech, 214−14), and Il-13 (Peprotech, 210−13) to establish fibrosis and M1/2 macrophage model, respectively. In brief, RAW264.7 cells at the seeding stage of Day 1 were pre-treated with LPS (1 μg/mL) [29] or IL4 (20ng/mL) combined with IL6 (20ng/mL) [30] for 6 hours, followed by exposure to 74DHF (100nM) [31] or VC (500μM) for 24 hours. The BEAS-2B cells were exposed to LPS (1 μg/mL) [32], TGF-β1 (5ng/mL) [33], 74DHF (100nM) or VC (500μM) for 24 hours. DMSO was used as vehicle control.

The CCK8 assay was performed based on the manufactural instructions. In brief, 1000 cells of RAW or BEAS2B were seeded in 96-well plate. The indicated dose of VC or 74DHF (100nM) was added on next day. After incubation for another 24 hours, the CCK8 solution was added and absorbance at 450 nm was measured.

### ELISA, transwell, PCR, and western blot (WB) analysis

The measurement of the inflammatory cytokines was performed using ELISA kits according to the manufacturer's instructions. The levels of TNF-α (4 A Biotech, CME0004) and IL-6 (4 A Biotech, CME0006) in the RAW264.7 cell culture supernatant were recorded. For the transwell assay, the BEAS-2B cells were seeded in transwell insert containing serum free medium (8μm, LABSELECT, 14341). The lower chamber contained complete medium with or without TGF-β1 (5ng/mL), 74-DHF (100nM) or VC (500μM). The invaded cells were counted after 24 hours culture by the staining of 0.1% crystal violet. The images were captured by a light microscope (DM6000B, Leica, Wetzlar, Germany) and quantified by the image J software (Wayne Rasband).

For the real-time PCR assay, the RNA extraction at the endpoint was performed by Trizol (Invitrogen, 1596−018) accordingly. We used the PrimeScript RT Reagent Kit with gDNA Eraser (TaKaRa) to perform the reverse transcription. The real-time PCR was performed by the ABI QS 12 RCR system with SYBR Green PCR Mix (Takara) using the primer below. *Tnf-α*: Forward-CCTGTAGCCCACGTCGTAG, Reverse- GGGAGTAGACAAGGTACAACCC, *Il-6*: Forward- CCAAGAGGTGAGTGCTTCCC, Reverse-CTGTTGTTCAGACTCTCTCCCT, *Arg-1*: Forward-CTCCAAGCCAAAGTCCTTAGAG, Reverse- GGAGCTGTCATTAGGGACATCA, *Muc5ac*: Forward- GTGGTTTGACACTGACTTCCC, Reverse-CTCCTCTCGGTGACAGAGTCT, *Cdh1*: Forward-CGACGGCAACTACAAGACCCG, Reverse-ACGAACTCCAGCAGGACCATG, *Acta2*: Forward- GGGCACTACCATGTACCCAG, Reverse- TGAAGGCGCTGATCCACAAAA.

The proteins were extracted from BEAS2B cells treated by TGF-β1 (5ng/mL) or indicated drugs for 48 hours. A total of 60 μg protein were electrophoresed on sodium dodecyl sulphate gel and transferred to PVDF membrane. The membrane was incubated with primary antibody against α-SMA (Abcam, ab7817), CDH1 (CST, 1065), COL1A1 (CST, 3395), AKT1 (CST, 2938), and GAPDH (Abcam, ab70699) over night. Next, the secondary HRP antibody was incubated for one hour at room temperature. The grey image was captured by Biorad chemidoc using ECL exposing kit and quantified by image J.

### Statistics

All experiments were replicated independently (N = 3). Data were analyzed by One-way analysis of variance (ANOVA) with Bonferroni's Multiple Comparison Test (means ± SEM).

## Results and discussion

### Potential synergistic effect of 74DHF and VC

**Fig 1:**
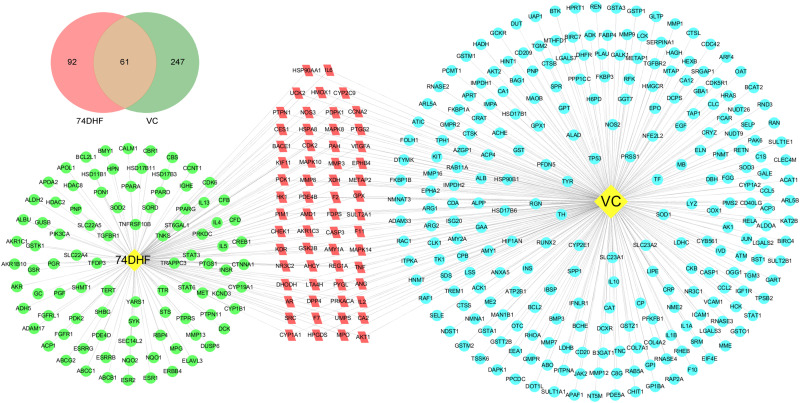
Venn diagram and C-T network of 74DHF and VC combination against asthma.

In the present work, to explore the synergetic mechanism of 74DHF and VC combination in the treatment of asthma, the common targets between 74DHF and VC were identified and analysed by Venn diagram and C-T network (Fig 1). The Venn diagram analysis showed that 153 targets of 74DHF and 308 targets of VC have 61 common targets, while the C-T network was constructed to reveal the complex interactions of them (data in S1 Text).

In Fig 1, 74DHF and VC shared 61 common targets, which are considered to be associated with the pathological processes of asthma. For instance, the evidence suggests that PTGS2 has been regarded as a key component in the inflammatory process of asthmatic airways and plays a pivotal role in the pathophysiological process of asthma [34]. SRC is reported to be necessary for airway smooth muscle cell growth and migration in airway remodeling, which is a potential therapeutic target for the treatment of asthma [35]. In addition, both 74DHF and VC possesses 92 and 247 specific protein targets respectively, that are notably associated with asthma. For instance, STAT3 as one of the target of 74DHF has contributed to influence the activation of the immune cells, thereby playing a key role in the progression of asthma [36]. For the specific target of VC, interleukin-1β (IL-1β) has been detected in bronchoalveolar lavage fluid, epithelial cells, and alveolar macrophages of asthmatic patients, suggesting that therapeutic strategies aimed at inhibiting IL-1β could potentially offer therapeutic advantages in managing asthma [37]. The findings indicate that a combination of 74DHF and VC exhibits a synergistic therapeutic impact on asthma through modulation of the intersecting and unique protein targets.

### Venn and PPI network analysis of 74DHF, VC and asthma

**Fig 2:**
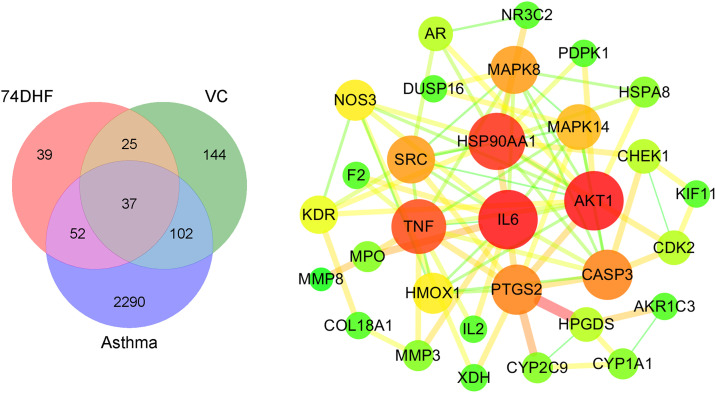
Overlapping targets and PPI network. **(A)**. The intersection of targets within 74DHF, VC, and asthma. **(B)**. The PPI network encompassing 37 intersecting targets.

To further elucidate the pharmacological therapeutic mechanism of 74DHF and VC combination against asthma, 153 and 308 potential targets of them were identified using four databases, including TCMSP, SEA, pharmGKB, pubchem (data in S2 Text). Moreover, we extracted 2481 asthma disease genes from the GeneCards database (data in S2 Text). After combining all targets and mapping them to the UniProt Database to confirm and convert into corresponding gene names specifically for Homo sapiens [25], a Venn diagram [26] was utilized to visually represent the intersecting genes among 74DHF, VC, and asthma (Fig 2A). Subsequently, a PPI network was constructed using STRING to delve deeper into the regulatory roles of the common targets (Fig 2B).

In Fig 2, the analysis focused on 37 shared targets between 74DHF (153), VC (308), and asthma (2481) through the Venn diagram. By employing STRING, a PPI network with 31 nodes and 83 edges was established to further investigate the complex interactions of these overlapped targets. 10 hub genes in PPI network exhibiting the highest coreness and betweenness were identified, including protein kinase B (AKT1), interleukin-6 (IL-6), heat shock protein 90 alpha family class A member 1 (HSP90AA1), tumor necrosis factor (TNF), caspase 3 (CASP3), prostaglandin-endoperoxide synthase 2 (PTGS2), proto-oncogene tyrosine-protein kinase src (SRC), mitogen-activated protein kinase 8 (MAPK 8), mitogen-activated protein kinase 14 (MAPK 14), heme Oxygenase 1 (HMOX1). Notably, these hub genes have been closely associated with asthma, indicating their potential importance as key targets of 74DHF and VC in the prevention and treatment of asthma. For instance, IL-6 as a multifunctional proinflammatory marker has been implicated in the pathogenesis of asthma due to its role in promoting systemic inflammation and airway remodeling [38]. It has been reported that TNF is a potent inflammatory cytokine that is thought to be important in immunoregulation of asthma by regulating airway inflammation and hyperresponsiveness [39]. These findings indicated that the hub target genes may have a significant impact on the pathological process of asthma for the combination of 74DHF and VC.

### GO processes enrichment of intersecting targets for 74DHF and VC on asthma

**Fig 3:**
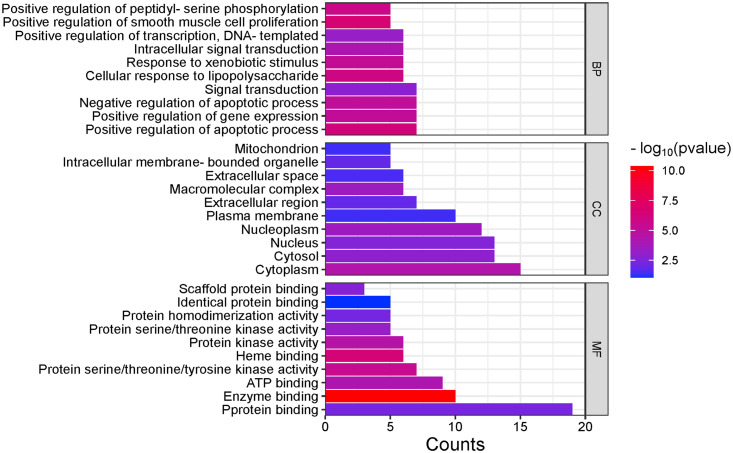
The distribution of GO entries in BP, CC, and MF of the intersecting hub targets among 74DHF, VC, and asthma. The number of genes in each category is represented by the height of the corresponding bar.

The GO enrichment analysis of intersecting targets for 74DHF, VC, and asthma was developed to further explore the potential synergistic mechanisms of 74DHF and VC against asthma. Three functional GO terms composed of BP, CC, and MF were enriched using ClueGO Cytoscape plugin (data in S3 Text). The top 10 GO terms of 20 intersecting targets for 74DHF, VC, and asthma involved in BP, CC, and MF were shown in Fig 3.

The top 10 BP terms suggested that 20 intersecting targets of 74DHF, VC, and asthma were primarily involved in positive regulation of apoptotic process, positive regulation of gene expression, negative regulation of apoptotic process, signal transduction, cellular response to lipopolysaccharide, response to xenobiotic stimulus, intracellular signal transduction, positive regulation of transcription, DNA-templated, positive regulation of smooth muscle cell proliferation, and positive regulation of peptidyl-serine phosphorylation. The top 10 CC with significant enrichment were cytoplasm, cytosol, nucleus, nucleoplasm, plasma membrane, extracellular region, macromolecular complex, extracellular space, intracellular membrane-bounded organelle, and mitochondrion. While the top 10 MF terms were relevant to protein binding, enzyme binding, ATP binding, protein serine/threonine/tyrosine kinase activity, heme binding, protein kinase activity, protein serine/threonine kinase activity, protein homodimerization activity, identical protein binding, and scaffold protein binding. Interestingly, these BPs, CCs and MFs are all linked to the pathological process and pharmacological mechanism of asthma. For instance, it is suggested that multiple signal transduction pathways have been linked to the inflammatory process in the airways of asthma patients, modulation of which may contribute to the design of new anti-inflammatory agents for asthma therapy [40]. In terms of CC, the relaxant response of β2-agonists has been suggested to bind with G-protein-coupled receptors and potassium channels expressed on the cell membrane of the airway smooth muscle cells which involve in the pathogenic mechanism of asthma [41]. For MF, the evidence indicates that protein kinases are novel identified factors in the pathological process of asthma, inhibition of which may be beneficial for the treatment asthma [42]. Collectively, the results revealed that 74DHF and VC may have a synergistic effect on asthma therapy through enriching the intersecting hub targets in these GO terms.

### Enrichment analysis of pathway for intersecting hub targets

**Fig 4:**
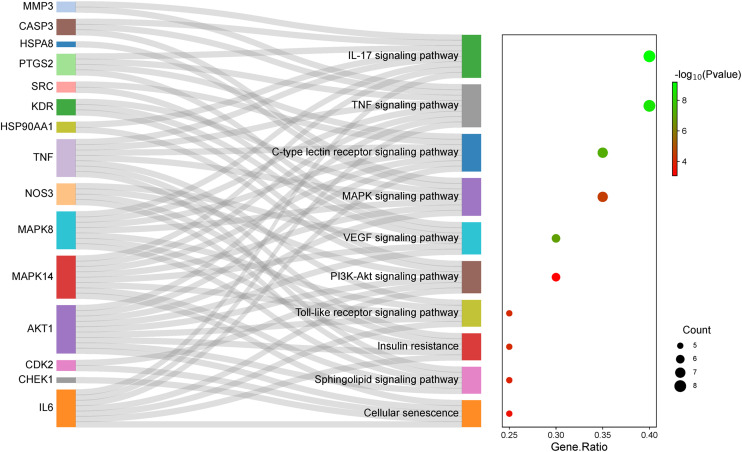
The Sankey bubble chart for the intersecting hub targets and pathways interactions.

The KEGG pathway enrichment analysis was performed to disclose the crucial signaling pathways of 74DHF and VC in the treatment of asthma using ClueGO Cytoscape plugin (data in S4 Text). The 20 intersecting hub targets among 74DHF, VC and asthma were mainly enriched in the 10 significantly pathways, including IL-17 signaling pathway, TNF signaling pathway, Toll-like receptor signaling pathway, PI3K-Akt signaling pathway, VEGF signaling pathway, MAPK signaling pathway, sphingolipid signaling pathway, insulin resistance, C-type lectin receptor signaling pathway, and cellular senescence (Fig 4). The 20 hub targets involved in these pathways showed more signiﬁcant characteristics. IL-17 signaling pathway showed the highest number of target associations (degree = 8) and are involved in the pathogenesis of asthma through regulating T-helper 2 (Th2) cell-mediated eosinophilic airway inflammation [43]. 7 targets participate in the MAPK signaling pathway, which may contribute to asthma by regulating the gene expression of inflammatory cytokines such as IL-6 [41]. Furthermore, it is reported that PI3K-Akt signaling pathway plays a role in a wide spectrum of asthma pathophysiology, which is regulated by 6 potential targets (IL6, HSP90AA1, NOS3, CDK2, KDR, AKT1) [44]. The discovered intersecting hub targets may exert a pharmacological effect against asthma through the regulation of diverse pathways. These findings may enhance our understanding of the synergetic mechanism of 74DHF and VC combination therapy for asthma from the pathway perspective.

### Validation of the anti-inflammatory and anti-fibrosis effects of 74DHF and VC

**Fig 5:**
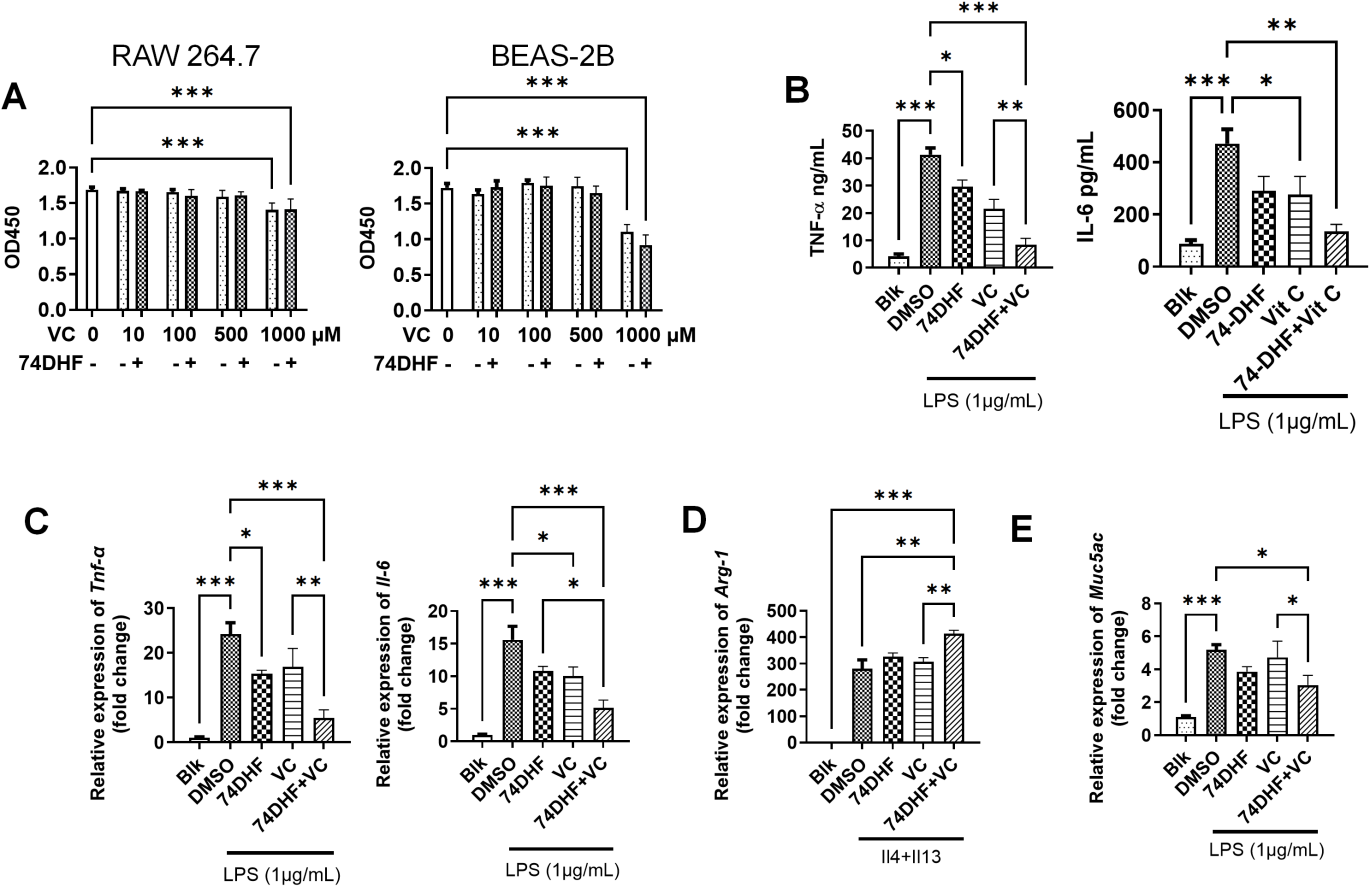
Validation of the anti-inflammatory effect of 74DHF and VC. **(A)**. CCK8 assay of RAW264.7 cell and BEAS2B cells treated with various doses of VC with or without 74DHF (100nM) (n = 5). **(B)** ELISA assay of the concentration TNF-α and IL-6 in the supernatants of RAW264.7 cell culture treated by LPS (1 μg/mL), 74DHF (100nM), or VC (500μM) (n = 3). **(C)**. Real time PCR analysis of *Tnf-α* and *Il-6* in RAW264.7 cells treated by 74DHF (100nM) or VC (500uM) under exposure of LPS (n = 3). **(D)** Real time PCR analysis of *Arg-1* in RAW264.7 cells treated by 74DHF (100nM) or VC (500uM) under exposure of IL4 (20ng.mL) and IL13 (20ng/mL) (n = 3). **(E)** Real time PCR analysis of *Muc5ac* in BEAS2B cells treated by 74DHF (100nM) or VC (500uM) under exposure of LPS (n = 3). Data are presented as the means ± SEM compared by one-way ANOVA with Bonferroni's Multiple Comparison Test. (*, *P* < 0.05, **, *P* < 0.01, ***, *P* < 0.001).

The present data suggested that the combined targets of 74DHF and VC were correlated with inflammatory pathways. Thus, we used the macrophage cell line RAW264.7 to evaluate the combined effects of these two chemicals on M1/2 macrophage (Fig 5A). Our data suggested that LPS induced about 10-fold and 20-fold upregulation in the TNF-α production at the protein (Fig 5B) and transcriptional level (Fig 5C), respectively. This augmentation was also observed in the IL-6 production, with 5-fold in protein level (Fig 5A) and 15-fold in transcriptional level (Fig 5B). Both 74DHF and VC treatment reversed the aberrant activation of TNF-α and IL-6 expression. VC showed more profound inhibition effect which was only observed in the TNF-α production compared to that from 74DHF (Fig 5B). Intriguingly, combined treatment showed synergistic effects on anti-inflammation (*P* < 0.01) which validated our *in silico* results. We further explored the M2 phase macrophage using IL4/13 induction. Consistently, VC and 74DHF improved the anti-inflammation function of M2 macrophage (Arg1). We also detected the key asthma marker Muc5ac, and the results showed that combination treatment significantly (*P* < 0.05) inhibited the Muc5ac induced by LPS treatment.

**Fig 6:**
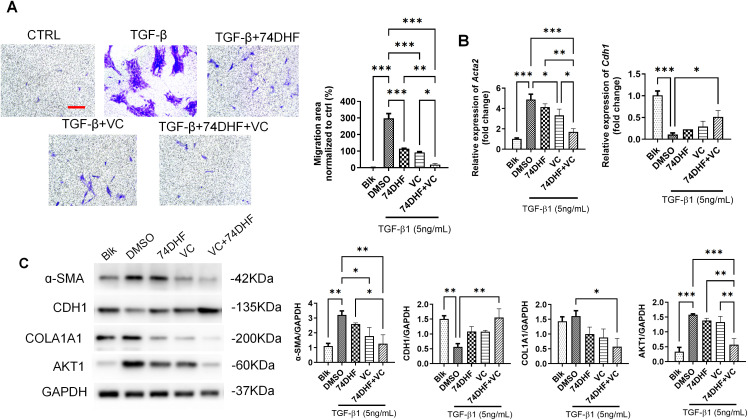
Validation of the anti-fibrosis effect of 74DHF and VC. **(A)**. Transwell analysis the migrated cells after TGF-β1 (5ng/mL), 74DHF (100nM), and VC (500μM) treatment. Scale bar: 100μm. The quantification of the stained cells was shown on the right (n = 3). **(B)**. Real time PCR of EMT marker *Acta2* and *Cdh1* expression in BEAS-2B cells treated by TGF-β1 (5ng/mL), 74DHF (100nM) or VC (500μM) (n = 3). **(C)** WB analysis of α-SMA, CDH1, COL1A1 and AKT1 in BEAS-2B cells treated by TGF-β1 (5ng/mL), 74DHF (100nM) or VC (500μM) for 24 hours (n = 3). The grey intensity was quantified on the right. Data are presented as the means ± SEM compared by one-way ANOVA with Bonferroni's Multiple Comparison Test. (*, *P* < 0.05, **, *P* < 0.01, ***, *P* < 0.001).

Moreover, SRC and PTGS2 were the hub genes shared by the targets of 74DHF and VC, which played a pivotal role in the epithelial to mesenchymal transition (EMT) related pulmonary fibrosis [45, 46]. Thus, we set out to test the addition of 74DHF and VC on the fibrosis in the bronchial epithelial cells BEAS-2B. The EMT and fibrosis were induced by TGF-β1 treatment. The transwell assay (Fig 6A) suggested that the 74DHF or VC significantly reduced the accelerated cellular migration caused by the TGF-β1 treatment (around 70%, *P* < 0.001). To note, the combined treatment of 74DHF and VC showed more obvious effects on the cellular invasion compared with 74DHF usage only (around 85%, *P* < 0.05). We went on to test the EMT and fibrosis related marks, cadherin 1 (CDH1) and alpha-smooth muscle actin (ACTA2). The transcriptional and WB analysis (Fig 6B and C) showed that both 74DHF and VC reversed the TGF-β1 induced ACTA2 up-regulation but only VC showed statistical significance (*P* < 0.05). Consistently, the combined treatment showed most significant effects on the downregulation of CDH1 and upregulation of ACTA2. Consistently, combination treatment inhibited the COLA1A expression under the exposure to TGF-β1. Taken together, 74DHF and VC showed synergistic effects on the inflammation and fibrosis which play a pivotal role in asthma. To note, the *in silico* model predicted the AKT1 to be a hub target which was also confirmed in the epithelial cell line (Fig 6C).

## Conclusions

Asthma is a complex and chronic inflammatory airway disease associated with a substantial global public health and economic consequences. 74DHF and VC have demonstrated pharmacological effects on asthma therapy respectively. However, there has been a paucity of research exploring their synergistic effects and associated mechanisms. Therefore, the current investigation employed a multifaceted systems pharmacology strategy encompassing target identification, data amalgamation, network pharmacology analysis, GO and pathway enrichment analysis, as well as *in vitro* experimental validation to delineate the underlying pharmacological action of the synergistic effect of 74DHF and VC in the treatment of asthma.

The key conclusions of this research can be summarized as follows. 153 and 308 potential targets of 74DHF and VC were identified, and 61 intersecting targets were screened between them. Then a comprehensive C-T network was established to explore their interaction mechanisms. Secondly, Venn diagram analysis and PPI network revealed 61 hub genes associated with asthma from the common intersection of 74DHF, VC, and asthma. The top 10 GO terms in BP, CC, and MF that were highly enriched in the combination therapy of 74DHF + VC for asthma, and these terms were found to be associated with 20 intersecting hub targets of 74DHF, VC, and asthma. Next, KEGG pathway enrichment analysis was conducted to uncover 10 crucial asthma-related pathways by utilizing the 20 intersecting hub targets, which provides insights into the pharmacological effects of 74DHF and VC combination in treating asthma from the pathway perspective. Last but not least, the *in vitro* experiments showed that 74DHF and VC have a synergistic effect on anti-inflammation in mice macrophages and fibrosis in human epithelium. To note, the regulatory pathways in inflammation and fibrosis should be validated in both models and even clinic to avoid the species-specific differences. The combined usage of these two chemicals ameliorated the LPS caused inflammation in macrophages and TGF-β caused EMT/fibrosis in lung epithelial cells.

In conclusion, the current study not only offers a thorough understanding of the intricate synergistic therapeutic mechanism of the combination of 74DHF and VC in asthma, but also presents a new prospect for the exploration of combination therapy in the management of asthma.

## Supporting information

S1 Text C-T- network. (XLSX)

S2 Text Target Name of 74DHF, VC, and Asthma. (XLSX)

S3 Text GO enrichment analysis. (XLSX)

S4 Text Pathway analysis. (XLSX)
